# Neuropsychiatric symptoms and executive function impairments in Alzheimer’s disease and vascular dementia: The role of subcortical circuits

**DOI:** 10.1590/1980-57642018dn13-030005

**Published:** 2019

**Authors:** Chan Tiel, Felipe Kenji Sudo, Ana Beatriz Calmon

**Affiliations:** 1 Universidade de Vassouras Medical School VassourasRJ Brazil Universidade de Vassouras (UV), Medical School, Vassouras, Rio de Janeiro, RJ, Brazil.; 2 Federal University of the State of Rio de Janeiro Department of Neurology Rio de JaneiroRJ Brazil Department of Neurology, Federal University of the State of Rio de Janeiro (UNIRIO), Rio de Janeiro, RJ, Brazil.; 3 Instituto D’Or de Ensino e Pesquisa Memory Clinic Rio de JaneiroRJ Brazil Memory Clinic, Instituto D’Or de Ensino e Pesquisa, Rio de Janeiro, RJ, Brazil.

**Keywords:** vascular dementia, Alzheimer, neuropsychiatric symptoms, apathy, demência vascular, Alzheimer, sintomas neuropsiquiátricos, apatia

## Abstract

Neuropsychiatric symptoms (NPS) in dementia are prevalent, under-recognized and little studied regarding their pathophysiological aspects. The pathophysiological mechanism, as well as the possible role of vascular lesions in the genesis of these symptoms, are still matters of debate.

**Objective:**

to describe and compare the prevalence and severity of NPS in subjects with Alzheimer's disease (AD) and vascular dementia (VaD).

**Methods:**

a cross-sectional study involving 82 outpatients, divided into two groups (AD × VaD), was conducted. Patients were submitted to the Cambridge Cognitive Test (CAMCOG), the Clock Drawing Test (CLOX 1 and 2), the Neuropsychiatric Inventory (NPI) and the Clinical Dementia Rating (CDR) scale. Neuroimaging was scored using the de Leon and Fazekas scales.

**Results:**

90.8% of the patients had at least one neuropsychiatric symptom. There were statistical differences on the CLOX test and in the apathy symptoms between AD and VaD groups. Apathy and disinhibition proved more prevalent in patients with higher vascular load.

**Conclusion:**

apathy and impaired executive function may reflect vascular damage in subcortical circuits in dementia patients.

## RESUMO

Sintomas neuropsiquiátricos na demência são prevalentes, pouco reconhecidos e ainda pouco estudados quanto aos aspectos fisiopatológicos. O mecanismo fisiopatológico, assim como o possível papel das lesões vasculares na gênese destes sintomas ainda são motivos de debate.

**Objetivo:**

descrever e comparar a prevalência e severidade dos sintomas entre a doença de Alzheimer (DA) e demência vascular (DV).

**Métodos:**

estudo transversal que incluiu 82 pacientes selecionados, divididos em dois grupos (DA × DV). Foram submetidos ao teste cognitivo de Cambridge (CAMCOG), teste do desenho do relógio (CLOX 1 e 2), inventário neuropsiquiátrico (INP) e avaliação clínica do estágio da demência (CDR). Avaliação dos índices de neuroimagem foram pelo escore de de Leon e Fazekas.

**Resultados:**

90.8% dos pacientes apresentavam pelo menos um sintoma neuropsiquiátrico. Houve diferença estatísticas no CLOX e sintoma apatia entre DA e DV. Apatia e desinibição demonstraram ser mais prevalentes nos pacientes com maior carga vascular.

**Conclusão:**

Apatia e alteração na função executiva podem refletir danos vasculares nos circuitos subcorticais naqueles pacientes com demência.

Dementia is a syndrome characterized by global acquired cognitive decline, which interferes in patients' activities of daily living.^1^^,^^2^ Although all available diagnostic criteria for this condition have been based on the presence of cognitive impairments, mounting

evidence recognizes that neuropsychiatric symptoms (NPS) are highly prevalent,^3^^-^7 highly detrimental to the patient's quality of life and strongly associated with caregiver burden.^8^^-^10 In addition, NPS may occur independently of the illness stage: in some cases, they may be the most prominent manifestation of the disorder; alternatively, they may precede cognitive changes^11^ or emerge only in moderate/severe stages of the disease.^8^^,^^12^ NPS commonly manifest as mood disorders, psychotic syndrome or other frontal features.^13^ More specifically, the most prevalent behavioral and mood symptoms in dementia are apathy, depression, agitation and irritability.^3^^,^^8^^,^^14^^,^^15^

Despite the importance of the issue for clinical practice, data on the pathophysiology of NPS is remarkably divergent or inconclusive in the literature. A cognitive hypothesis for behavioral changes has been formulated, implying that underlying impairments in cognitive functions may be associated with NPS. For instance, apathy may correlate with executive function problems,^16^ whereas a possible relationship between global cognitive impairment and anxiety has been indicated by some authors.^17^ Nonetheless, heterogeneities in the methods used to evaluate these symptoms could account, to some degree, for the disparities in findings across studies.^4^^,^^5^^,^^6^^,^^18^ Notably, the overlap of Alzheimer's disease (AD) and Vascular Dementia (VaD) pathologies in samples may hamper researchers' ability to draw conclusions about the mechanisms underlying these clinical features.

The Neuropsychiatric Inventory (NPI) is possibly the most used instrument to investigate NPS in research and clinical practice.^19^^,^^20^ It assesses, through a brief structured interview applied to the patient's caregiver, the frequency and severity of twelve domains of symptoms.^19^ It has been translated into Brazilian Portuguese and validated for the Brazilian elderly population.^21^

The goal of this study was to compare NPI scores in patients with Alzheimer and Vascular disease. We also sought to investigate whether severity of vascular lesions correlated with NPS and cognitive performance in our sample.

## METHODS

### Participants

A cross-sectional study including eighty-two patients with Alzheimer and Vascular dementia, recruited at outpatient healthcare facilities in the state of Rio de Janeiro, Brazil, between May of 2017 and May of 2018 was conducted.

Selection of participants was based on the following eligibility criteria: (1) subjects were diagnosed as presenting AD or VaD according to the DSM-5 criteria (American Psychiatric Association, 2013); (2) individuals were older than 59 years old; (3) subjects agreed to undergo a complete medical investigation, including physical examination, neuroimaging and laboratory tests; and (4) patients had no current or past history of severe psychiatric and neurological disorders, substance abuse, traumatic brain injury or exposure to neurotoxic agents.

### Procedures

Initial assessment of participants comprised a clinical interview and physical evaluation, including neurological examination. Subsequently, a set of cognitive screening tests were applied to the sample: the Mini-Mental State Examination (MMSE),^22^ Cambridge Cognitive Test (CAMCOG)^23^ and the Clock Drawing Test (CLOX method).^24^ The Clinical Dementia Rating (CDR) scale was employed to determine the staging of cognitive impairment.^25^ The Neuropsychiatric Inventory (NPI) was applied to an informant to measure the presence and severity of NPS.^21^

In addition, brain Magnetic Resonance Imaging (MRI) exams were evaluated if field strengths were at least 1.5 T and scans had been conducted within 1 year prior to cognitive testing. Severity of white-matter hyperintensities was visually analyzed by a trained neurologist using the modified-Fazekas scale (mF).^26^ Concomitantly, the degree of hippocampal atrophy was rated using the de Leon scale.^27^

### Ethics

The study was approved by the Research Ethics Committee of the Federal University of Rio de Janeiro State (UNIRIO) under the protocol 79326117.1.0000.5258.

### Data analysis

The Kolmogorov-Smirnov test was used to measure the normality of data. Mean differences between diagnostic groups (AD and VaD) were determined using Student's *t*-test for normally distributed continuous variables, while Mann- Whitney's U-test was used in other cases. Pearson's Chi Square was employed to evaluate distributions of categorical variables across groups.

Additionally, subjects were clustered according to mF, and mean differences in continuous variables were analyzed by ANOVA and Tukey's post-hoc test (when variables followed normal distribution) or Kruskal-Wallis test. Partial correlations were used to assess associations among behavioral and cognitive variables and mF (analyzed as an ordinal variable), controlling for severity of hippocampal atrophy (as measured by the De Leon scale). Linear Logistic Regression was conducted to verify the predictive relationship of cognitive and behavioral aspects with white-matter damage. To this end, items significantly correlated with mF were included as independent variables and mF was defined as the dependent variable. The level of significance was set at p <0.05. The IBM Statistical Package for the Social Sciences (SPSS) v. 25 was used for data analyses.

## RESULTS

In this study, 82 older adults were included (mean age =72.97±8.84 years, range = 60-91 years; education = 5.50±3.54 years, range = 0-16 years; 54.9% female participants). Concerning diagnoses, 41 patients were classified as presenting AD and 41 had VaD. Prevalence of NPS in the whole sample was 90.8% of participants.

AD subjects were significantly older than VaD patients (p<0.001). No differences between groups were found for education or global cognitive performance (CAMCOG scores). However, subjects with AD performed significantly better on the CLOX 1 and 2 than VaD subjects (p<0.001). The VaD cluster scored significantly higher on the NPI Apathy item than the AD cluster (p<0.001). None of the other NPS domains significantly distinguished the groups. These findings are shown in Table 1.

**Table 1:** Differences between diagnostic groups for continuous sociodemographic, cognitive and behavioral variables.

Variable	Group	N	Mean	Standard deviation	p-value
Age	AD	41	76.46	7.76	<0.001*
	VD	41	69.48	8.55	
Education	AD	41	5.80	3.79	n.s.*
	VD	41	5.19	3.28	
CAMCOG	AD	41	56.58	8.31	n.s.*
	VD	41	56.82	8.39	
CLOX1	AD	41	9.63	1.31	<0.001*
	VD	41	7.04	1.68	
CLOX2	AD	41	10.02	1.44	<0.001*
	VD	41	7.90	1.64	
Delusion	AD	41	.17	.70	n.s.**
	VD	41	.75	1.71	
Hallucination	AD	41	.82	1.98	n.s.**
	VD	41	.73	1.44	
Agitation	AD	41	1.87	2.63	n.s.**
	VD	41	1.36	2.49	
Depression	AD	41	.48	1.32	n.s.*
	VD	41	2.82	3.47	
Anxiety	AD	41	1.12	1.79	n.s.**
	VD	41	1.04	2.60	
Euphoria	AD	41	.29	1.05	n.s.**
	VD	41	.04	.31	
Apathy	AD	41	1.60	2.71	<0.001*
	VD	41	4.82	3.55	
Disinhibition	AD	41	.19	.74	n.s.**
	VD	41	.26	.80	
Irritability	AD	41	1.34	2.25	n.s.**
	VD	41	1.09	2.60	
Motor	AD	41	.29	1.05	n.s.**
	VD	41	.48	2.22	
Sleep	AD	41	1.21	2.47	n.s.**
	VD	41	1.07	2.32	
Appetite change	AD	41	.53	1.55	n.s.**
	VD	41	.87	2.27	
NPI	AD	41	9.97	7.55	n.s.**
	VD	41	15.31	13.23	

* Student's *t*-test;

** Mann-Whitney

As expected, AD patients had lower scores on the mF and higher scores on the De Leon scale than VaD subjects, as shown in Table 2. CDR scores did not differ between groups.

**Table 2:** Distribution of categorical variables between the diagnostic groups.

Variables		AD	VD	p-value
Sex	Male	16	21	n.s.
	Female	25	20	
mF	0	20	2	<0.001
	1	21	11	
	2	0	22	
	3	0	6	
Hippocampus	0	0	16	<0.001
	1	16	20	
	2	18	5	
	3	7	0	
CDR	0	0	0	n.s.
	0.5	0	0	
	1	27	24	
	2	14	17	
	3	0	0	

When the sample was clustered according to mF scores, the group with no white-matter hyperintensities (mF=0) differed significantly on the NPI Apathy subtest compared to those with severe white-matter impairments. Disinhibition score was higher in subjects with mF=3 than in those with moderate white-matter hyperintensities (mF=2). No cognitive or other behavioral variables distinguished mF groups (Table 3).

**Table 3:** Sociodemographic, clinical/cognitive and NPI staging score data.

Variables	mF=0	mF=1	mF=2	mF=3	p-value
Age	73.0±18.38	65.18±7.40	70.18±8.35	73.66±6.86	n.s.*
Education	6.00±2.82	5.36±3.38	5.45±3.44	3.66±2.94	n.s.*
Gender (M/F)	0/2	8/3	10/12	3/3	n.s.**
CDR (0/0.5/1/2/3)	0/0/1/1/0	0/0/7/4/0	0/0/14/8/0	0/0/2/4/0	n.s.**
Hippocampus (0/1/2/3)	1/1/0/0	6/4/1/0	6/13/3/0	3/2/1/0	n.s. **
CAMCOG	65.00±4.24	57.09±8.14	57.09±9.39	52.66±2.94	n.s.*
CLOX 1	8.50±0.70	7.90±1.75	6.77±1.65	6.00±0.89	n.s.*
CLOX 2	9.50±0.70	8.18±1.99	7.77±1.57	7.90±1.64	n.s.*
Delusion	0±0	0.72±1.61	0.50±1.05	2.00±3.34	n.s.^#^
Hallucination	0±0	0.54±1.29	0.95±1.70	0.50±0.83	n.s.^#^
Agitation	0±0	1.45±3.69	1.50±2.08	1.16±1.83	n.s.*
Depression	3.00±4.24	2.27±3.22	2.36±3.04	5.50±4.80	n.s.*
Anxiety	0±0	1.36±3.10	0.72±1.45	2.00±4.89	n.s.^#^
Euphoria	0±0	0±0	0.90±0.42	0±0	n.s.^#^
Apathy	0±0	4.27±3.28	4.68±3.09	8.00±4.17	mF=0≠mF=3*
Disinhibition	0±0	0.18±0.60	0.09±0.42	1.16±1.60	mF=2≠mF=3^#^
Irritability	0±0	1.63±3.88	1.04±2.19	0.66±1.63	n.s.^#^
Motor	0±0	0±0	0.54±2.55	1.33±3.26	n.s.^#^
Sleep	0±0	0.90±2.07	1.09±2.36	1.66±3.20	n.s.^#^
Appetite Change	0±0	0.36±1.20	1.27±2.84	0.66±1.23	n.s.^#^
NPI total	3.00±4.24	13.36±15.40	14.86±10.50	24.66±16.88	n.s.*

* ANOVA and Tukey's *post-hoc*;

** Pearson's Chi-square;

# Kruskal-Wallis.

After controlling for scores on the De Leon scale, performance on the CLOX 1, Apathy and Disinhibition moderately correlated with mF (Table 4). These variables were included in the Linear regression Model, using mF as the dependent variable. Apathy and CLOX 1 were predictors of severity of vascular burden.

**Table 4:** Correlations between mF and behavioral and cognitive variables, after controlling for degree of hippocampal atrophy.

Variable	Correlation mF	p-value
CAMCOG	n.s.	-
CLOX 1	-0.41*	p<0.01
CLOX 2	n.s.	-
Delusions	n.s.	-
Hallucinations	n.s.	-
Agitation	n.s.	-
Depression	n.s.	-
Anxiety	n.s.	-
Euphoria	n.s.	-
Apathy	0.40*	p<0.05
Disinhibition	0.32**	p<0.05
Irritability	n.s.	-
Motor	n.s.	-
Sleep	n.s.	-
Appetite Change	n.s.	-
NPI total	n.s.	-

^a^Pearson's Partial correlation; ^b^Spearman's Partial Correlation.

## DISCUSSION

The present study indicated that NPS are present in over 90% of subjects with dementia, a rate in line with previous reports.^6^^,^^14^^,^^15^ Of the NPS domains, apathy appeared to be strongly correlated with the diagnosis of VaD and might reflect higher severity of subcortical vascular damage. Our data also supports the idea that performance on the CLOX 1, a measure of executive control,^24^ may reflect degree of vascular burden.

Integrity of frontal and subcortical interconnections has been deemed crucial for executive function, as assessed by the CLOX 1.^28^ In fact, this task may identify subcortical impairment even in early stages, according to a previous study, which might be explained by the complexity of this cognitive function, in terms of the brain regions potentially recruited during executive tasks.^29^ For instance, the ability to draw a clock without visual stimuli might be dependent on the coordinated activation of visuospatial, memory and motor control-related cortices, as well as of prefrontal areas responsible for planning, abstract thinking and working memory.^30^ It could be inferred that vascular-related disconnection of one or more of these long subcortical inter-lobe fibers, which allow this combined activity of brain areas, may affect executive function.^31^

Previous reports describe a higher prevalence of apathy in VaD compared to AD.^32^^-^35 This symptom could be described as loss of initiative and motivation.^21^ Akin to executive function, apathy might stem from disconnections of frontal-subcortical regions. Consistently, studies have shown that damage to the uncinate fasciculus can lead to loss of interconnections among prefrontal areas, amygdala and the fusiform gyrus, which could result in the onset of apathy.^36^^-^38 Moreover, cholinergic pathways, which are spread widely throughout the subcortex, may also be disrupted in small vessels disease.^32^^,^^33^ Evidence has suggested a role of those circuits for motivational drive;^39^ therefore, lesions to the cholinergic system may also result in apathy.

Disinhibition is classified as impulsive hypersexualized behavior, activities or comments, with an excess of jocosity and even weird or inappropriate behavior. The presence of this clinical feature also distinguished AD from VaD in our sample; however, mean scores on this NPI domain were low, implying that these symptoms were not prominent in our sample. Difficulties in social cognition and inhibitory control, which are also related to the prefrontal cortex, were associated with this finding in some studies.^40^

The results of this study highlight the importance of vascular load in the severity of apathy and disinhibition. Similar data were found in previous studies investigating apathy in VaD,^32^^,^^33^ but are not reported in studies related to VaD and disinhibition. The evidence in the present study may point to a possible influence of white-matter hyperintensities on the genesis of disinhibition. Therefore, better control of cardiovascular risk factors is of paramount importance for health in late-life.

Some limitations of this study should be highlighted. Significant age differences were evident between AD and VaD patients. However, this finding, which might be interpreted as a potential confounding factor regarding cognitive and neuroimaging variables, could correspond to some aspects of these disorders in real life. Neurodegenerative diseases may be more prevalent in advanced age groups,^1^ whereas VaD, at least the “pure” presentations, might be more prevalent in younger subjects.^41^^,^^42^ Hence, the attempts to control for or match age between groups could have impacted the ecological validity of our findings. Moreover, the cross-sectional design of the study precluded further inferences, especially regarding prevalence of NPS. In addition, it has been recognized that most subjects with dementia may present both AD and VaD pathology in the brain, which cannot be detected using MRI and may affect the ability to draw conclusions when comparing groups. Finally, as observed in most informant-based instruments, the NPI may have inherent biases. Cultural issues and the presence of caregiver burden, or even the neuropsychiatric symptom itself, may affect answers on the instrument. Therefore, responses might exhibit underestimation or overestimation of the true situation.^10^^,^^12^

In conclusion, the authors suggest that measures of apathy and executive function, namely the CLOX, can accurately distinguish VaD from AD. A possible role of frontal-subcortical disconnection could be relevant to both phenomena.
